# F1000 Workspace

**DOI:** 10.5195/jmla.2017.9

**Published:** 2017-01

**Authors:** Erica R. Brody, Kathleen A. McGraw, Barbara Rochen Renner

## BACKGROUND

F1000 Workspace (F1000W) is a relative newcomer to the reference database scene, joining the ranks of EndNote, RefWorks, Mendeley, Papers, and Zotero. In 2015, the London-based Science Navigation Group launched F1000W, the third of a suite of products originally branded as the Faculty of 1000 with the goal of “Changing the way science is communicated” [1]. The first of this trio of products, F1000 Prime, was released in 2002 to provide expert recommendations about the best research articles in biology and medicine. Following in 2012, F1000 Research is an open access, open science journal that facilitates rapid publication of life sciences findings with transparent post-publication peer review.

As the latest F1000 addition, F1000W combines reference management with powerful tools to support collaborative scholarly writing. It provides space to gather and organize references with attached portable document format files (PDFs), along with any related manuscripts in development, into private or group projects. Manuscripts are tracked by robust version control that includes documentation of the author and time stamps of all edits. Similarly, F1000W documents all database activity, including reference additions and deletions, as well as annotations of records and full-text articles. These features make F1000W stand out in the crowded field of similar tools for its strong support of collaborative work on literature review and manuscript development. Other highlights include unlimited storage space, word processing plug-ins, and exceptional customer support characterized by rapid response times, helpful information, and sincere interest in feedback about the program’s functionality.

This review highlights the features that support the work of health sciences librarians. Comments are based on the experience of two health sciences librarians and a library science student using F1000W through mid-August 2016.

## SYSTEM REQUIREMENTS

F1000W is a web-based program accessible from any Internet-connected device, but full functionality is only available with a desktop or laptop computer. A lightweight desktop application allows users to upload PDFs or folders of PDFs directly to their reference libraries. The company is planning to release mobile-friendly apps for Android and iOS in fall 2016.

The program works with all of the major web browsers, including Chrome, Firefox, Internet Explorer 11, Opera, and Safari 6 onwards. The desktop application can be uploaded onto systems with either Windows (XP or later) or OS X for Mac (version 10.7 onwards).

## ACCESS

Individual or institutional subscribers can set up an account at f1000.com/work/. Users can associate their accounts with an institution, which includes links to full-text articles in F1000W via their organizations’ subscriptions. Each account has unlimited storage capacity; however, the system will not upload individual PDFs larger than 100 megabytes.

**Figure 1:**
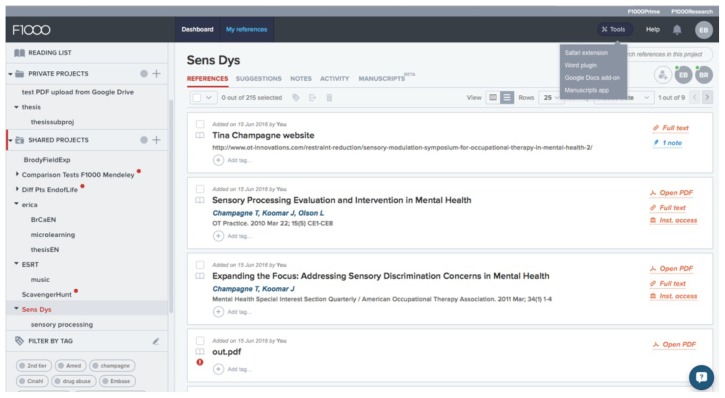
F1000 Workspace user interface

To access all capabilities, users download the program’s plug-ins located in the “Tools” menu (Figure 1) before importing references. Extensions are available for all major browsers that allow users to import citations directly from literature searches in most databases, including PubMed, CINAHL, and Scopus. In addition, add-ons for Microsoft Word, Google Docs, and Manuscripts (an iOS app) allow users to embed citations in manuscripts.

## CITATION MANAGEMENT

### Importing references and full-text articles

Users can add references and the full text of articles to F1000W in several ways. Users can enter citations manually; retrieve and import citations via unique identifiers such as digital object identifier (DOI), PubMed ID (PMID), or International Standard Book Number (ISBN); use a browser extension to add citations directly from reference databases and journal websites; or import files of references saved in Research Information Systems (RIS), BIB, NBIB, and Endnote extensible markup language (XML) file formats. Individual online PDFs can be imported via a browser extension. PDFs stored on the computer, Google Drive, or Drop-Box can be imported individually or in folders. Users can also specify a “watch folder,” after which any PDFs in that folder will be automatically uploaded to the reference library. F1000W reliably extracts citation data from PDFs that have DOIs, while older PDFs or those obtained via scanning of print documents will not generate this metadata. Once a project is populated with several references, F1000W displays “Suggestions,” a list of recommended articles from PubMed based on the system’s analysis of the titles, abstracts, and authors of the articles in that project [2]. Suggested articles recommended by F1000 Prime are presented at the top of the list. Users may provide feedback regarding the relevance of each suggestion, which refines the algorithm for identifying suggested articles.

### Handling missing data

F1000W designates references as “Missing Data” when the system suspects a citation is incomplete. The Edit Metadata button on the detailed reference view offers users the option to use a DOI or PMID to retrieve additional metadata or to edit the metadata manually. When viewing a PDF in F1000W, the Fix Metadata feature offers users a list of potential article records that may match the reference requiring additional data.

### Finding duplicates

Duplicates are identified based on all references that users have stored in the system rather than references associated with a specific project. F1000W screens for duplicates during the import process and retains the version with the most complete information. To the reviewers’ knowledge, detailed research on the accuracy of duplicate removal through merger has not been conducted for this tool. We compared several sample projects with duplicate removal using EndNote and had similar results with fewer steps in F1000W. Users can also find and merge duplicates. When references already in “All References” are imported into a project that does not have them, that record is also displayed in the project. Changes made to a reference accessed in one folder or project are displayed when users view the record in any other folder where it is stored.

### Organizing, sharing, and annotating references

F1000W offers several tools for organizing, sharing, and annotating references: folders, tags, and notes. F1000W displays four standard folders: All Records, Unsorted, Missing Data, and Reading List. In addition, users can create projects and subprojects. Projects may be private or shared with other users; subprojects inherit the membership of their parent projects. All user activity, such as adding and deleting references or notes, can be viewed for each project and subproject by clicking the project’s Activity tab. Records can also be organized using tags, which remain private even when they are assigned to records in a shared project. Tags can be assigned to individual records or batches of records. Users can add notes—shared or private—directly to a record or can create notes by highlighting and annotating PDFs. All PDF annotations are color-coded to indicate the user who made them.

### Searching references

The search feature at the top right of the screen allows users to search across references in the currently selected project. Search results include all instances of a word or phrase in the citation information and annotations but not tags associated with references. There is no advanced search with options to retrieve results from a particular field, but records can be sorted by field.

### Exporting references

F1000W exports references in BibTeX, RIS, or CSV file types. Most other citation management programs recognize at least one of these formats, so an F1000W library can be exported into another database. The CSV option allows users to import all reference, tag, project, and subproject information into Microsoft Excel, with the exception of annotations. The RIS export format permits importing into Covidence or RevMan for systematic review. PDF files can be downloaded separately from references but do not contain the highlights or annotations added in F1000W.

## SYSTEMATIC REVIEW MANAGEMENT

By planning the structure of projects and subprojects and using standardized language for tags and notes that are unique to the project folders, systematic review teams could use F1000W to document the selection of articles by multiple reviewers. The source database of each reference can be tracked by importing the results from each database into a separate subproject. The top-level project folder will contain the merged results of all database searches. The ability to add both public and private notes, as well as private tags, allows both blind review and communication between reviewers.

## BIBLIOGRAPHY CREATION: CITING AND WRITING

When writing in Microsoft Word, Google Docs, and the iOS Manuscripts app, users can search for citations using keywords, author, or title, or filter references by tag or project, and F1000W will generate a list of potential citations for insertion. While writing offline, users may note citation information (e.g., author name) within curly braces. Once reconnected to the Internet, the “Find citation marks” button generates a list of potential citations from users’ libraries, Pub-Med, or recommended articles that can be selected and inserted into the document.

The Search PubMed and Smart Citation Suggestions buttons allow users to search for references in PubMed or from a list of suggested PubMed citations. Users who would like to cite a source that is not in their F1000W library can also save the citation information to their F1000W library from within the word processing software.

The Bibliography button allows users to select a citation style and insert a bibliography. Searching among available styles is quite easy: users can search by name of style or example of citation format. Users cannot edit styles; however, the F1000W staff will create a new style upon request (within twenty-four hours, based on our experience).

The Export function creates a plain-text version of the document without the F1000W field codes attached to the references, as may be required by a publisher for manuscript submission. The Share With Co-Authors feature uploads the document into the Manuscripts section of a project in F1000W (distinct from the Manuscripts app). Files stored in Manuscripts can be reviewed and commented on in F1000W, as well as edited by multiple users simultaneously in Microsoft Word. F1000W tracks the author and timing of the different versions of the document for precise version control.

Also in Microsoft Word, Submit to F1000 Research allows users to submit their papers to F1000 Research. After publishing an initial article in F1000 Research for free, authors pay processing charges between $150 and $1,000, based on article length and size of associated data files.

## LIBRARIAN FEATURES

Institutional account holders can obtain metrics about F1000W use at their organization upon request: total users accessing from this institution; users accessing for the first time from this institution; new projects with a member at this institution; existing, active projects with a member at this institution; total projects with a member at this institution; references added by this institution’s users; and total references in this institution’s projects.

## F1000W SUPPORT

F1000W includes a comprehensive, searchable user’s guide accessible from any page in the interface. Support is also available via chat, with a response promised within one business day.

## LIMITATIONS OF F1000W

When evaluating F1000W for adoption, users should consider the following limitations of the program. As a web-based program, F1000W requires an Internet connection. In addition, uploading multiple references to the database can be slow. Unlike several of its competitors, F1000W does not show abstracts or notes in a preview pane when displaying lists of references. Users have to click into the details of a reference for this information, which slows down the process of reviewing references. Organization of references is limited to two levels of project, and tags cannot be viewed among shared project members.

## RECOMMENDATIONS

F1000W is a robust citation management database that effectively supports manuscript creation by one or more authors in three different word processing programs. For those who are familiar with reference management software, the program’s intuitive interface is easy to learn. The comprehensive help documentation and responsive technical support further ensure users’ ability to take full advantage of the program’s features. The tool might not be ideal for those in developing countries or locations where Internet availability is intermittent or limited. The product is best suited to research in most areas of biology and medicine, as F1000W supports seamless integration with F1000 Prime and Pub-Med.

User feedback fuels the evolution of F1000W functionality, so recommendations submitted to the technical support staff may lead to improvements.
